# Factor Xa Generation by Computational Modeling: An Additional Discriminator to Thrombin Generation Evaluation

**DOI:** 10.1371/journal.pone.0029178

**Published:** 2012-01-11

**Authors:** Kathleen E. Brummel-Ziedins, Thomas Orfeo, Matthew Gissel, Kenneth G. Mann, Frits R. Rosendaal

**Affiliations:** 1 Department of Biochemistry, University of Vermont, Burlington Vermont, United States of America; 2 Departments of Clinical Epidemiology and Thrombosis and Haemostasis, Leiden University Medical Center, Leiden, Netherlands; Leiden University Medical Center, Netherlands

## Abstract

Factor (f)Xa is a critical enzyme in blood coagulation that is responsible for the initiation and propagation of thrombin generation. Previously we have shown that analysis of computationally generated thrombin profiles is a tool to investigate hemostasis in various populations. In this study, we evaluate the potential of computationally derived time courses of fXa generation as another approach for investigating thrombotic risk. Utilizing the case (n = 473) and control (n = 426) population from the Leiden Thrombophilia Study and each individual's plasma protein factor composition for fII, fV, fVII, fVIII, fIX, fX, antithrombin and tissue factor pathway inhibitor, tissue factor-initiated total active fXa generation was assessed using a mathematical model. FXa generation was evaluated by the area under the curve (AUC), the maximum rate (MaxR) and level (MaxL) and the time to reach these, TMaxR and TMaxL, respectively. FXa generation was analyzed in the entire populations and in defined subgroups (by sex, age, body mass index, oral contraceptive use). The maximum rates and levels of fXa generation occur over a 10- to 12- fold range in both cases and controls. This variation is larger than that observed with thrombin (3–6 fold) in the same population. The greatest risk association was obtained using either MaxR or MaxL of fXa generation; with an ∼2.2 fold increased risk for individuals exceeding the 90^th^ percentile. This risk was similar to that of thrombin generation(MaxR OR 2.6). Grouping defined by oral contraceptive (OC) use in the control population showed the biggest differences in fXa generation; a >60% increase in the MaxR upon OC use. FXa generation can distinguish between a subset of individuals characterized by overlapping thrombin generation profiles. Analysis of fXa generation is a phenotypic characteristic which may prove to be a more sensitive discriminator than thrombin generation among all individuals.

## Introduction

The inventory of blood and vessel wall components associated with the blood coagulation system for hemorrhage control is extensive, and this inventory increases substantially with the inclusion of intermediate species that emerge in the individual processes leading to thrombin generation. Monitoring thrombin generation has been the focus of providing a global description of the blood coagulation process, due to the multifactorial nature of thrombin in procoagulant, anticoagulant, fibrinolytic and cellular events [1]. Thrombin's critical role in maintaining hemostasis is also exemplified by the use of antithrombotics to suppress coagulation. Most of these agents target thrombin, platelets and, more recently, factor (f)Xa [2].

Factor Xa can be thought of as the transducer of the thrombin generation signal. The main function of fXa is to participate in the prothrombinase complex (fXa-fVa-membrane-Ca^2+^). Factor Xa is the serine protease enzyme in the prothrombinase complex that catalytically activates prothrombin to thrombin. Factor Xa is a unique regulatory enzyme in that it is formed through both the extrinsic tenase (tissue factor (Tf)-fVIIa-membrane-Ca^2+^) and intrinsic tenase (fVIIIa-fIXa-membrane-Ca^2+^) complexes. During the initial stages of the hemostatic event triggered by the exposure of blood to Tf, low levels of both fXa and fIXa are generated [3]. Once generated, the limited amounts of fXa produced by the extrinsic tenase bind to available membrane sites and convert picomolar amounts of prothrombin to thrombin [4]. This thrombin then activates fVIII and fV allowing the initial formation of the intrinsic tenase and prothrombinase complexes. The burst or propagation phase of thrombin generation depends upon the additional fXa generated via the intrinsic tenase complex. The intrinsic tenase complex activates fX at a 50- to 100- fold higher rate than the extrinsic tenase complex [5]–[7]. This increased rate of fXa generation overcomes the suppressive action of fXa inhibitors such as tissue factor pathway inhibitor (TFPI) and antithrombin (AT) resulting in increasing levels of prothrombinase and the propagation of the procoagulant event. It has also been shown, that the prothrombinase concentration is limited by the active fXa concentration [8], [9]. Thus, the termination of prothrombinase activity is primarily a consequence of the inhibition of fXa. Because fXa is a major player in the coagulation process, it has been a target for regulation by synthetic inhibitors in treating ischemic heart disease and cerebrovascular disease. Many studies are currently underway to clinically evaluate antithrombotic agents that target fXa [10].

Prothrombin circulates in plasma at a concentration of 1.4 µM (100 µg/mL) while the plasma concentration of circulating fX is approximately 10-fold less (∼170 nM, 10 µg/mL). Studies from a number of experimental systems have indicated that as a coagulation reaction proceeds, consumption of fX is quite limited (<10%) in contrast to prothrombin [11]. The practical consequence of this has been that measuring fX consumption or fXa levels in coagulation reactions is technically more challenging than monitoring prothrombin or thrombin products. There are currently no widely accessible methods comparable to those that directly monitor thrombin formation, such as thrombograms, or that indirectly measure thrombin, such as thrombin-antithrombin (TAT) complex measurements.

Factor X levels in healthy populations vary on average over a two- to three-fold range. High levels of fX alone have been shown to predict the risk of venous thrombosis, but were not a risk factor for venous thrombosis when the levels of other vitamin K-dependent proteins were taken into account [12]. Little is known regarding the levels of fXa that are generated in healthy individuals upon a tissue factor (Tf) stimulus, and how variations in fXa may effect thrombin generation and the overall procoagulant response leading to DVT. Methods that enable the evaluation of fXa generation in individuals might add to our understanding of variations in thrombin generation among individuals and might help guide the use of fXa anticoagulants.

Mathematical modeling allows for these measurements and comparisons. An empirically validated numerical model of the extrinsic coagulation system [13]–[17] provides a method for investigating fXa generation for profiles and patterns in a large group of individuals. In this study, we used a mathematical model that describes the Tf pathway of blood coagulation to evaluate fXa generation in the Leiden Thrombophilia Study (LETS) cohort and compare the results to previously analyzed thrombin generation in this population [15]. This is the first study that we are aware of that compares fXa generation to thrombin generation in the same population using computational modeling approaches.

## Materials and Methods

### Study population

Our population was selected from the LETS case-control study [18]. The healthy controls (n = 474) were sex and age matched acquaintances or partners of patients within the case group (n = 474, patients with an objectively diagnosed first deep vein thrombosis). All individuals were younger than 72 years and did not have active malignancy. Individuals on oral anticoagulation (n = 49) were excluded from these analyses, leaving 473 controls and 426 patients.

Several individual characteristics were used to define subpopulations. We separated the data by sex, age and body mass index. When we investigated the effect of oral contraceptives we excluded individuals who were pregnant, post-menopausal, within 30 days postpartum or had a recent miscarriage at the time of thrombosis for patients (index date). Since the median time between occurrence of DVT and venipuncture was 18 months, we only evaluated women who either used oral contraceptives at the index date and the venipuncture date or did not at both dates. Seven individuals were excluded from analyses involving alcohol since information was missing, and three individuals were excluded from analyses involving BMI who were not 18 at the time of the blood draw. Factor Xa generation curves of each subpopulation were generated by averaging fXa concentrations at each time point. Subpopulation fXa profiles are shown in the figures as the mean time point values with the 95% confidence interval.

### Plasma composition

Citrated plasma previously collected from all individuals [18] was used to determine the concentrations of the coagulation proteins fII, fV, fVII, fVIII, fIX, fX, TFPI and AT as described in detail in earlier studies performed using the LETS population (described in [15]). In brief, fII activity was measured by a chromogenic assay. Factor V, fIX, fX and TFPI were determined by antigen based assays. Factor VII and fVIII levels were measured by one-stage coagulation assays. Antithrombin was measured by a colorometric assay (Coamate, Chromogenix, Mölnda, Sweden) [19]. All procoagulant and anticoagulant protein levels were determined for each individual and are shown as the mean (SD) and range in Table 1.

**Table 1:** Plasma composition within either healthy controls or individuals with a known DVT.

	Controls: No Known DVT		Prior DVT	
Protein	PercentageMean (SD)	Range	PercentageMean (SD)	Range
**FII**	104 (15)	63–153	108 (17)	67–178
**FV**	131 (33)	47–302	133 (35)	41–305
**FVII**	110 (22)	41–171	114 (25)	53–200
**FVIII**	122 (33)	49–232	141 (35)	53–318
**FIX**	103 (21)	52–188	109 (26)	63–209
**FX**	103 (17)	49–163	107 (20)	58–174
**AT**	99 (10)	63–125	99 (11)	67–143
**TFPI total**	92 (21)	46–171	94 (21)	35–159

### Numerical simulations

**Figure 1:**
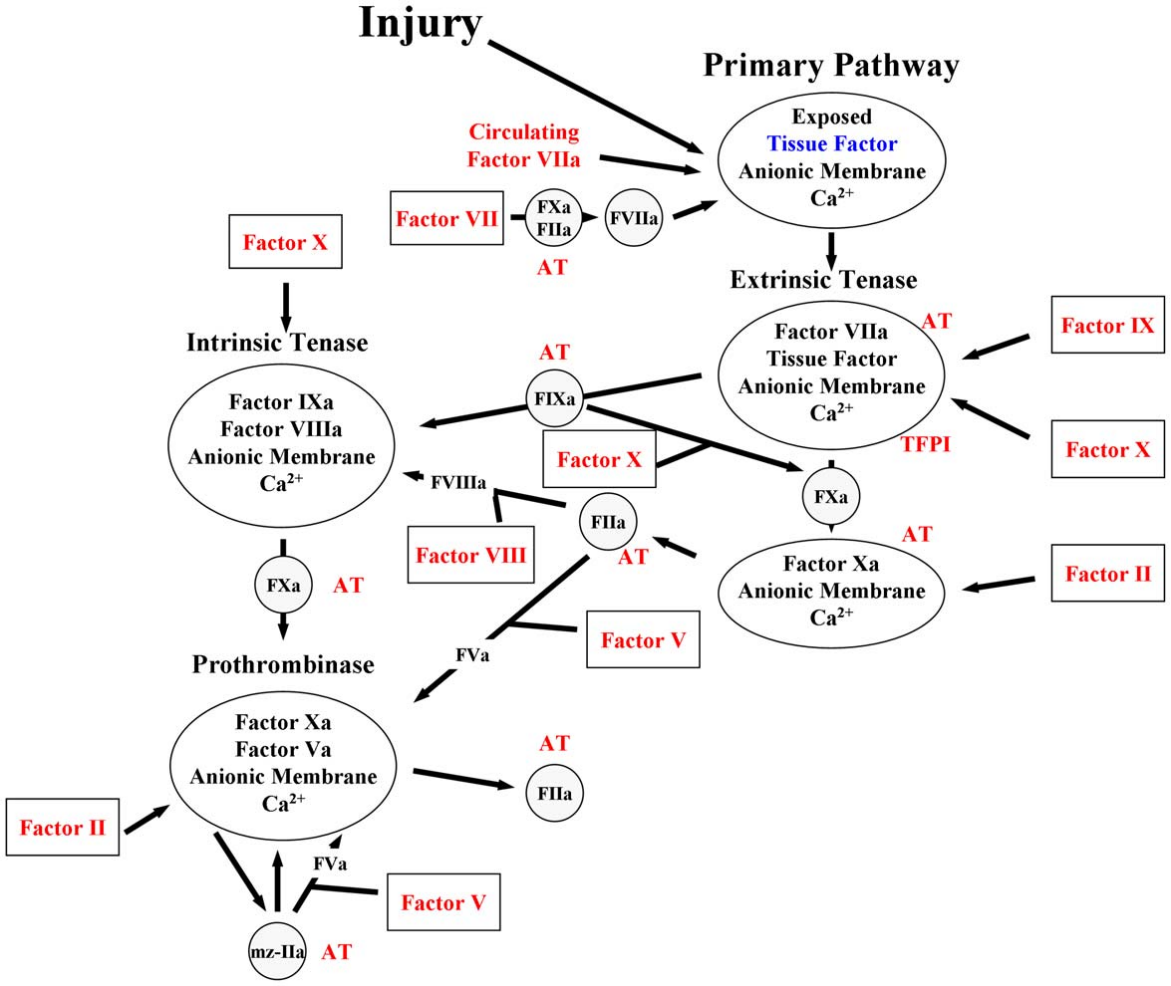
Schematic of the computational model of the tissue factor pathway. The multicomponent processes encompassed in the computational model are illustrated as either: enzymes (open circle), inhibitors (hatched circle), zymogens (open boxes) or complexes (open ovals). Upon injury to the vessel wall, tissue factor, the cofactor for the extrinsic tenase complex, is exposed to circulating factor VIIa and forms the vitamin K dependent complex the extrinsic tenase. Factor IX and factor X are converted to their serine proteases factor IXa (FIXa) and factor Xa (FXa) which then form the intrinsic tenase and the prothrombinase complexes, respectively. The combined actions of the intrinsic and extrinsic tenase and the prothrombinase complexes lead to an explosive burst of the enzyme thrombin (IIa). The procoagulant response is down regulated by the stoichiometric inhibitors tissue factor pathway inhibitor (TFPI) and antithrombin (AT). TFPI serves to attenuate the activity of the extrinsic tenase trigger of coagulation. AT directly inhibits thrombin, FIXa and factor Xa. The proteins in which the concentrations from individual subjects are used are shown in red. Tissue factor in blue is arbitrarily set to 5 pM. Circulating FVIIa is set at 1% of the concentration of FVII from each individual.

The mathematical model is based upon prior publications by Hockin *et al*. [13] and Butenas *et al*. [14] and yields concentration versus time profiles for selected species when electronic mixtures of procoagulant (fII, fV, fVII/fVIIa, fVIII, fIX and fX) and anticoagulants (TFPI and AT) are exposed to a 5 pM Tf stimulus (Figure 1). Factor levels expressed as a percentage were translated into molar concentrations by using literature values for the mean plasma concentrations [20]. Each individual's plasma factor composition was entered into the computer database, and simulated reactions were initiated with Tf. Simulated reactions were solved for total active fXa generation (free fXa and fXa in the prothrombinase complex produced by both the extrinsic and intrinsic tenase complexes) over a 3600 second time frame. The outputs of these fXa profiles were evaluated by the maximum level (MaxL-Xa) and rate (MaxR-Xa) of fXa generated, the time to reach these values (TMaxL-Xa and TmaxR-Xa) and total fXa (summing fXa concentrations at each time point: AUC).

A systematic analyses of the contribution of the plasma factors to either the fXa or thrombin generation output was conducted on the subgroups, as previously described [21], [22], by adjusting the factors to mean physiologic concentrations or to the relevant control population and reevaluating the output.

### Statistical analyses

Data are presented as mean (SD). The Kolmogorov-Smirnov test was used to assess conformity with a normal distribution. The t-test was utilized for comparisons between two groups provided a normal distribution. Non-parametric methods were utilized if the assumption of non-normality was not met. Significance was defined as p<0.05. 90^th^ percentiles for MaxL, MaxR and AUC were determined for the entire population. Odds ratios (signifying the odds of being a case for those above the 90^th^ percentile vs those with lower levels) were calculated for each parameter.

## Results

### Factor Xa generation

**Figure 2:**
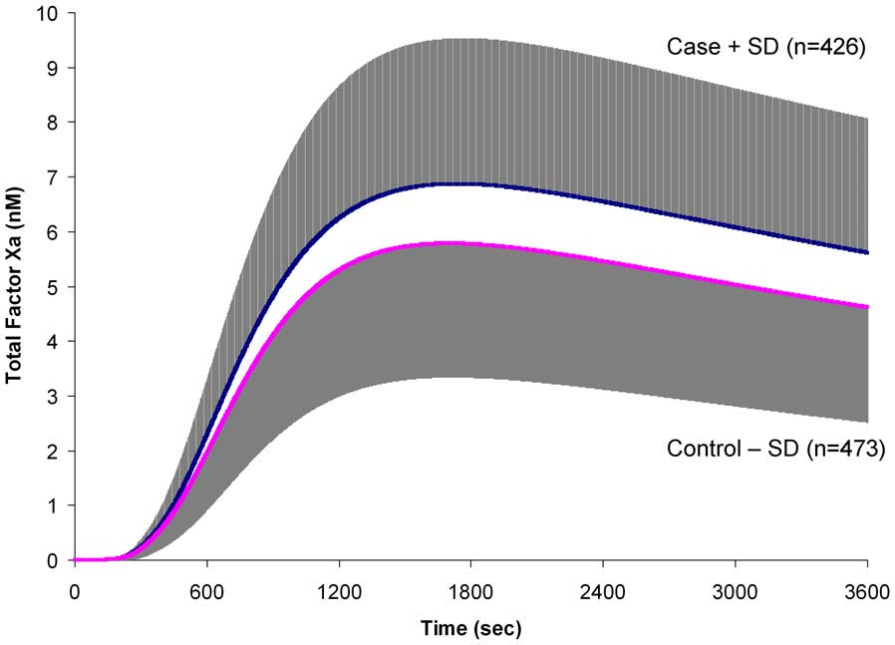
Factor Xa profiles of case and control individuals. Plasma compositions from 473 healthy individuals and 426 individuals with a known DVT were used to generate time courses of total active fXa upon a Tf stimulus of 5 pM. The mean total active fXa level at each second is shown with the standard deviation in grey.

The fX activation profiles for all cases and controls are represented separately in Figure 2 as a mean (SD) profile. The extracted parameters for MaxR, TMaxR, MaxL, TMaxL and AUC are presented in Tables 2 and 3. The MaxR-Xa generation varied within the healthy control population by 12-fold (2.3–28 pM/s; IQR: 5.6–9.5 pM/s; P10: 4.3 pM/s; P90: 12.4 pM/s) and the MaxL-Xa varied over 10-fold (1.7–17 nM; IQR: 4.0–6.9 nM; P10: 3.1 nM; P90: 9.0 nM). Within the same control population using the same mathematical model, the range of thrombin (IIa) generation was tighter for both the MaxR-IIa (1.1–5.4 nM/s; IQR: 2.0–2.9 nM/s; P10: 1.7 nM/s; P90: 3.6 nM/s) and the MaxL-IIa (158–669 nM; IQR: 313–414 nM; P10: 275 nM; P90: 462).

**Table 2:** Factor Xa generation outputs for healthy controls (no known DVT).

	N	MaxR,pM/smean (SD)	TMaxR,smean (SD)	MaxL,nMmean (SD)	TMaxL,smean (SD)	AUCµM * smean (SD)
**Whole population**	473	8.0 (3.5)	636 (31)	5.8 (2.5)	1737 (203)	15.5 (6.7)
**Sex**						
**Men**	201	7.2 (2.8)†	647 (29)†	5.3 (2.1)†	1769 (178)†	14.2 (5.7)†
**Women**	272	8.5 (3.9)	629 (30)	6.2 (2.7)	1714 (216)	16.3 (7.2)
**Females without OC**	90	7.2 (2.3)†	633 (27)†	5.2 (1.7)†	1677 (153)	13.7 (4.7)†
**Females with OC**	47	12.0 (5.4)	601 (27)	8.4 (3.5)	1630 (198)	22.2 (9.4)
**Age (yrs)**						
**≤45**	223	7.9 (3.9)	633 (33)‡	5.7 (2.7)	1678 (158)†	15.0 (7.2)
**>45**	250	8.0 (3.1)	639 (28)	5.9 (2.2)	1790 (223)	15.9 (6.1)
**BMI kg/m^2^**						
**≤26**	264	7.5 (3.4)†	638 (32)	5.4 (2.2)†	1699 (173)†	14.2 (6.0)†
**>26**	193	8.6 (3.6)	633 (29)	6.4 (2.6)	1789 (227)	17.2 (7.1)

N: population size; BMI: Body Mass Index, OC: oral contraceptives, MaxR: maximum rate, TMaxR: time to maximum rate, MaxL: maximum level, TMaxL: time to maximum level, AUC: area under the curve.

† p≤0.001 when compared within each stratification,

‡ p<0.05.

**Table 3:** Factor Xa generation outputs for individuals with a known DVT.

	N	MaxR,pM/smean (SD)	TMaxR,smean (SD)	MaxL,nMmean (SD)	TMaxL,smean (SD)	AUCµM * smean (SD)
**Whole population**	426	9.4 (3.6)	634 (28)	6.9 (2.7)	1776 (243)	18.5 (7.4)
**Sex**						
**Men**	172	8.6 (3.2)†	643 (26)†	6.4 (2.3)‡	1828 (265)†	17.1 (6.3)‡
**Women**	254	9.9 (3.8)	627 (27)	7.3 (2.9)	1741 (221)	19.4 (7.9)
**Females without OC**	40	10.1 (3.8)‡	626 (21)†	7.7 (3.0)‡	1797 (206)	20.5 (8.3)
**Females with OC**	30	12.6 (5.1)	605 (21)	9.3 (4.0)	1711 (260)	25.0 (11.3)
**Age (yrs)**						
**≤45**	209	9.3 (3.7)	627 (27)†	6.8 (2.8)	1739 (269)‡	18.1 (7.7)
**>45**	217	9.4 (3.5)	640 (27)	7.0 (2.6)	1812 (210)	18.8 (7.1)
**BMI kg/m^2^**						
**≤26**	199	8.8 (3.4)‡	634 (30)	6.4 (2.5)†	1734 (229)†	17.1 (6.7)†
**>26**	216	9.9 (3.7)	633 (26)	7.4 (2.8)	1819 (252)	19.9 (7.7)

N: population size; BMI: Body Mass Index, OC: oral contraceptives, MaxR: maximum rate, TMaxR: time to maximum rate, MaxL: maximum level, TMaxL: time to maximum level, AUC: area under the curve.

† p≤0.001 when compared within each stratification,

‡ p<0.05.

Within the case population (individuals with a known DVT), MaxR-Xa generation also varied by 12-fold (of 2.1–25.1 pM/s) and MaxL-Xa and AUC varied over 11-fold (1.7–18.6 nM and 4.5–51.8 µM*s, respectively). In comparison to thrombin generation in the case population, fXa discrimination was consistently broader than thrombin generation; which showed only a 5.8 fold variation in MaxR-IIa (11.3–65.3 nM/s) and 5.3 fold in MaxL-IIa (170–903 nM) [21]. When evaluated by the odds ratio at a 90^th^ percentile cut off, the OR for MaxR-fXa generation is 2.1 (95% CI: 1.3–3.2) and for MaxL-fXa generation is 2.2 (95% CI: 1.4–3.4) (Table 4). Thrombin generation MaxR had an OR of 2.6 (95% CI: 1.8–3.8) at the same cut off point. The fXa generation parameters MaxR (95% CI for difference: 0.93–1.86 pM/s), MaxL (95% CI for difference: 0.77–1.44 nM) and AUC (95% CI for difference: 2.075–3.925 µM*s) were significantly greater in the cases.

**Table 4:** Comparison of fXa generation in individuals with a known DVT versus controls.

		N	MaxRPrevalence, %	MaxLPrevalence, %
**Whole population**	Controls	473	33 (7.5%)	32 (7.3%)
	Cases	426	57 (15.4%)	58 (15.8%)
	Fisher's Exact p-value		0.0017	0.0008
	OR (95% CI)		2.1 (1.3–3.2)	2.2 (1.4–3.4)
**Sex/Men**	Controls	201	7 (3.5%)	7 (3.5%)
	Cases	172	15 (8.7%)	14 (8.1%)
	Fisher's Exact p-value		0.046	0.070
	OR (95% CI)		2.6 (1.1–6.7)	2.5 (0.97–6.2)
**Sex/Women**	Controls	272	26 (9.6%)	25 (9.2%)
	Cases	254	42 (16.5%)	43 (16.9%)
	Fisher's Exact p-value		0.019	0.009
	OR (95% CI)		1.9 (1.1–3.2)	2.0 (1.2–3.4)
**Females without OC**	Controls	90	0 (0%)	1 (1.1%)
	Cases	40	6 (15%)	6 (15%)
	Fisher's Exact p-value		0.0006	0.0034
	OR (95% CI)		undefined	15.7 (1.8–135)
**Females with OC**	Controls	47	14 (29.8%)	13 (27.6%)
	Cases	30	11 (36.7%)	12 (40.0%)
	Fisher's Exact p-value		0.62	0.32
	OR (95% CI)		1.4 (0.52–3.6)	1.7 (0.66–4.6)
**Age≤45 yrs**	Controls	223	17 (7.6%)	17 (7.6%)
	Cases	209	31 (14.8%)	32 (15.3%)
	Fisher's Exact p-value		0.021	0.015
	OR (95% CI)		2.1 (1.1–3.9)	2.2 (1.2–4.1)
**Age>45 yrs**	Controls	250	16 (6.4%)	15 (6.0%)
	Cases	217	26 (12.0%)	26 (12.0%)
	Fisher's Exact p-value		0.051	0.032
	OR (95% CI)		2.0 (1.0–3.8)	2.1 (1.1–5.6)
**BMI≤26 kg/m^2^**	Controls	264	12 (4.5%)	10 (3.8%)
	Cases	199	20 (10.1%)	18 (9.0%)
	Fisher's Exact p-value		0.026	0.029
	OR (95% CI)		2.4 (1.1–4.9)	2.5 (1.1–5.6)
**BMI>26 kg/m^2^**	Controls	193	20 (10.4%)	21 (10.9%)
	Cases	216	35 (16.2%)	38 (17.6%)
	Fisher's Exact p-value		0.11	0.066
	OR (95% CI)		1.7 (0.93–3.0)	1.8 (0.99–3.1)

OR: odds ratio calculated at the 90% cut off point; BMI: body mass index; OC: oral contraceptives.

### Factor Xa generation segregated by clinical risk categories

Factor Xa generation in individuals was analyzed by grouping fXa generation profiles of individuals with shared clinical characteristics (*e.g*. BMI, sex, age, oral contraceptive use) and generating an average profile for the entire group analogous to our previously published work for thrombin [15], [21]. The results are summarized in Tables 2 and 3 and described below.

### Body mass index

**Figure 3:**
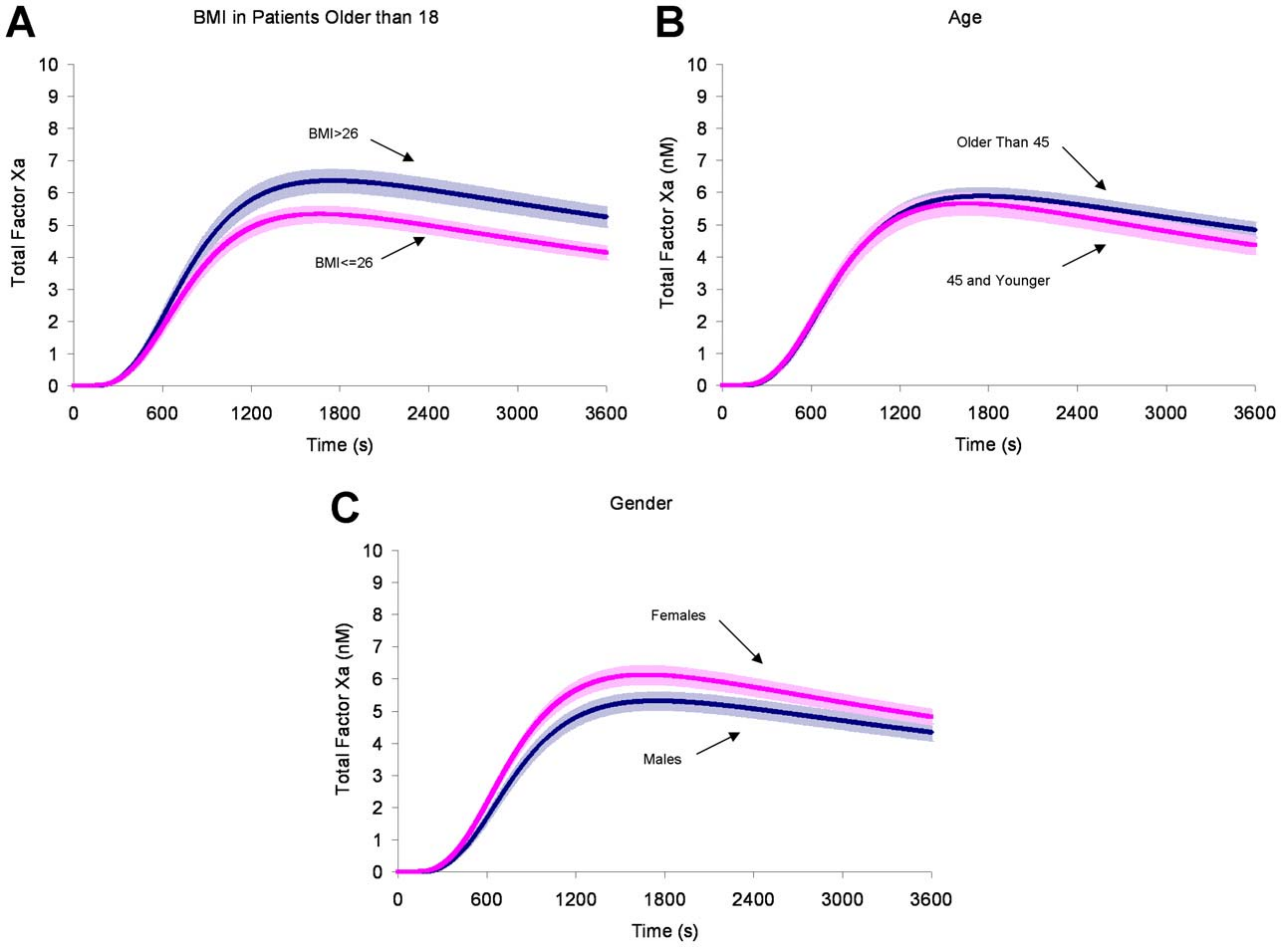
Factor Xa generation segregated by potential risk factors. Individuals within the healthy control population were separated into categories to evaluate the response of fXa generation between groups. The groups were: **Panel A**) Body Mass Index (BMI); ≤26 kg/m^2^ (n = 264) and >26 kg/m^2^ (n = 193); **Panel B**) Sex; men (n = 201) and women (n = 272); **Panel C**) Age; ≤45 yrs (n = 223) and >45 yrs (n = 250). All fXa profiles are shown as the mean and the 95% CI.

Figure 3 panel A presents the computational analyses of fXa generation when the control population is segregated into individuals with either a BMI ≤26 kg/m^2^ or a BMI >26 kg/m^2^. This BMI segregation reflects the one used in the original thrombin generation analysis [15], [21]. In the control group, for every 1 unit increase in BMI, there was an average of a 0.138 pM/s increase in MaxR (95% CI: 0.0577–0.2181), a 13 sec increase in TML (95% CI: 8.516–17.528), a 0.372 µM*s increase in AUC (95% CI: 0.223–0.522) and 0.128 nM increase in MaxL (95% CI: 0.0726–0.1832).

When cases and controls are compared (Table 4), individuals with a lower BMI (≤26 kg/m^2^) showed an odds ratio of 2.4 (95% CI: 1.1–4.9) for MaxR and 2.5 (95% CI: 1.1–5.6) for MaxL. Thrombin generation, as previously reported, also showed a greater risk prediction (MaxR OR: 2.9 (95% CI: 1.6–5.0)) in individuals with a lower BMI ≤26 kg/m^2^ [21]. However, unlike thrombin, fXa generation for individuals whose BMI >26, both MaxR and MaxL had 95% CIs encompassing 1.

### Age

Figure 3 panel B presents the computational analyses of fXa generation when the control population is segregated into age categories of individuals >45 years old or 45 years old and younger. In the control population, for every 1 year increase in age, there was an average of a 0.381 sec increase in TMR (95% CI: 0.172–0.591), and a 5.02 sec increase in TML (95% CI: 3.70–6.34). MaxR, MaxL and AUC were not different. Both age groups showed an elevated odds ratio for cases for both MaxR and MaxL (Table 4). This was also true when thrombin generation as a risk predictor was evaluated [21].

### Sex and oral contraceptive use

When control women (n = 272) are compared to men (n = 201) (Figure 3C), women generate fXa at a faster rate (8.5 (3.9) pM/s versus 7.2 (2.8) pM/s; 95% CI for difference: 0.7–1.9 pM/s) and reach higher levels (6.2 (2.7) nM versus 5.4 (2.1) nM; 95% CI for difference: 0.4–1.2 nM). (Table 2).

**Figure 4:**
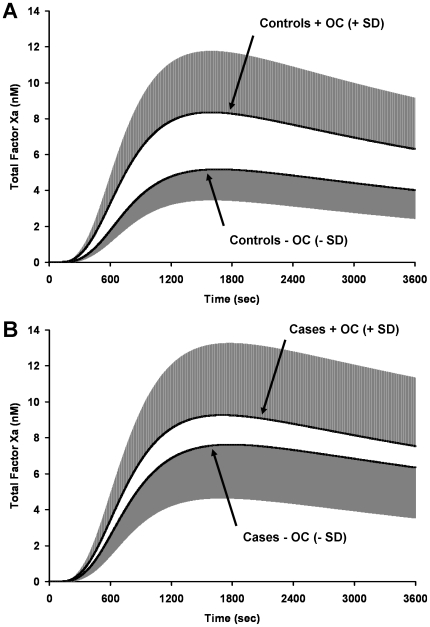
Oral contraceptive use. Premenopausal women aged 15 to 49 were segregated into categories of: **Panel A:** control women +OC use (n = 47) and –OC use (n = 90) and **Panel B:** women with a known DVT +OC use (n = 30) and –OC use (n = 40). FXa generation was evaluated for these groups. The grey shaded area represents 1 SD.

When control premenopausal women aged 15 to 49 are split into users and non-users of oral contraceptives (Table 2), we observe the largest disparity in fXa generation among the clinical risk categories that we evaluated (Figure 4A). When women on oral contraceptives (n = 47) were compared to women not on oral contraceptives (n = 90), fXa was generated at a faster MaxR (12.0 (5.4) pM/s versus 7.2 (2.3) pM/s; 95% CI for difference: 3.1–6.4 pM/s) and a higher MaxL (8.4 (3.5) nM versus 5.2 (1.7) nM; 95% CI for difference: 2.2–4.3 nM) in women who took oral contraceptives. The time to reach the maximum rate was significantly faster in users (601 (27) s versus 633 (27) s; 95% CI for difference: 23–42 s). In addition, more total fXa was generated over the course of the experiment (22.2 (9.4) vs. 13.7 (4.7) µM*s; 95% CI for difference: 5.6–11.4 µM*s). Thrombin generation was also markedly enhanced in women who used oral contraceptives [21].

For this select group of premenopausal women not on oral contraceptives, when women with a known DVT are compared to control women, the OR for MaxL of fXa generation is 15.7 and for MaxR is undefined since no controls (out of 90 individuals) were above the 90^th^ percentile compared to cases (6 out of 40 were above the cut off, Table 4). Thrombin generation measurements for MaxR were similar in that no control individuals were present at a 90^th^ percentile cut-off point [21].

### Comparing factor Xa generation in control individuals with equivalent thrombin generation profiles

**Figure 5:**
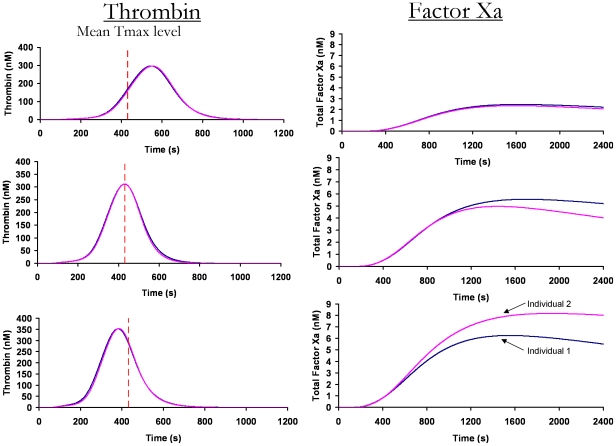
Comparing factor Xa and thrombin generation. Thrombin generation profiles from the same population, previously determined using the same methodology [15], are compared to their fXa profiles. Three pairs of overlapping individual thrombin generation profiles are shown on the left. The dashed line indicates the mean time to the maximum level of thrombin generation within the healthy population (432 s). The corresponding fXa generation profiles in the same individuals are shown on the right.

To determine if fXa generation provides any additional discrimination between individuals relative to thrombin generation, we reevaluated our previously determined thrombin generation profiles [15] within the same control population. We selected three pairs of thrombin generation profiles in which each of the individual profiles within a pair were overlapping (Figure 5). The first pair of individuals had overlapping thrombin profiles that were generated more slowly (∼120 s slower) than the mean time to maximum level (dashed line, 432 s) of the healthy population. The second pair of individuals yielded overlapping thrombin generation curves comparable to the mean profile (dashed line). The third pair of individuals yielded overlapping thrombin generation curves that were faster (∼60 s faster) than the mean profile.

When we evaluate fXa generation in the individuals with overlapping pairs of thrombin generation curves, fXa generation appears to discriminate between the individuals in two of the groupings. There also appears to be a relationship between the timing of thrombin generation and the level of fXa discrimination. The greatest discrimination appears when the overlapping thrombin generation profiles are faster than the mean of the population (bottom row). These results show that fXa generation can provide additional discrimination to thrombin generation profiles. In addition, these studies were performed with a Tf-initiator concentration of 5 pM to reflect empirically validated data [13]. Additional discrimination is possible when Tf is evaluated at varying Tf concentrations; further studies are warranted to elucidate this effect.

We have previously demonstrated that the thrombin generation profile is not dependent upon one factor alone, but the synergy between all the factors [15]. We have shown that small differences in the ensemble of factor levels, all within the normal range, can lead to large differences in thrombin generation profiles [15], [16], [21], [22]. In this case, the focus is on the opposite effect. That is, that when considerably different normal range ensembles are evaluated with respect to thrombin generation potential, no difference in thrombin generation is predicted. For example, the plasma composition between the two individuals with the faster than normal overlapping thrombin generation curves are as follows: individual 1: fII 97%, fV 89%, fVII 157%, fVIII 137%, fIX 87%, fX 134%, AT 99%, TFPI 64%; individual 2: fII 97%, fV 164%, fVII 131%, fVIII 188%, fIX 100%, fX 124%, AT 101%, TFPI 87%. Striking differences in factor levels include fV (80% difference), fVIII (35% difference) and TFPI (35% difference). Although these thrombin profiles are virtually identical, when the dynamics are evaluated using a different parameter (fXa) the two individuals can be distinguished.

### Identifying the compositional differences that drive the alterations in fXa generation profiles

**Figure 6:**
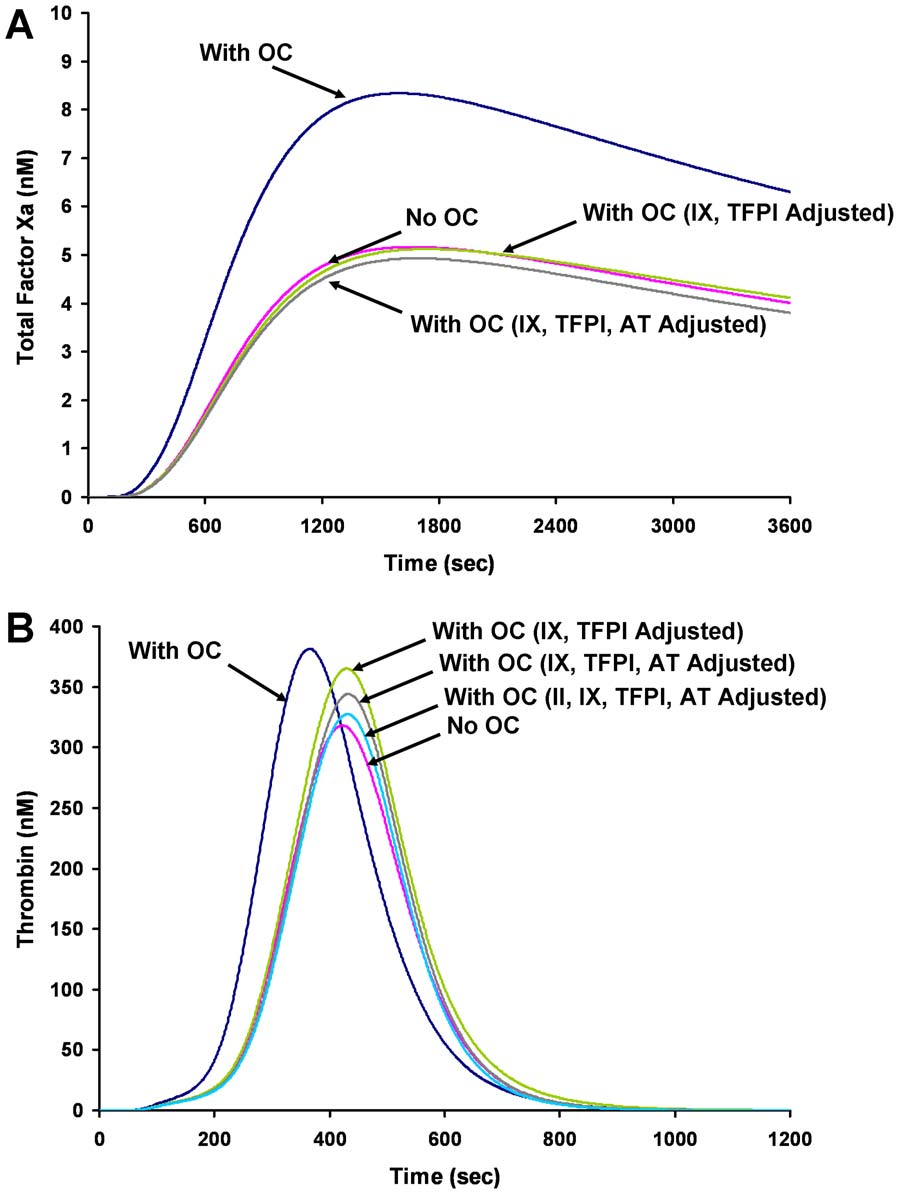
Factor Xa and thrombin generation curves for oral contraceptive users and non-users. **Panel A:** Factor Xa curves for oral contraceptive users, non-users, users with fIX and TFPI adjusted to the mean value of the non-users and users with fIX, TFPI and AT adjusted to the mean value of the non-users. **Panel B:** Thrombin curves for oral contraceptive users, non-users, users with fIX and TFPI adjusted to the mean value of the non-users, users with fIX, TFPI and AT adjusted to the mean value of the non-users and users with fIX, TFPI, AT and fII adjusted to the mean value of the non-users.

We have reported differences in thrombin generation profiles between acute (ACS) and stable coronary artery disease (CAD) populations [22] and have determined that in the ACS population, the procoagulant phenotype seen with thrombin generation profiles appears to depend primarily on the influence of AT, fVIII and prothrombin. A similar analysis was performed here to identify which protein(s) account for differences in fXa profiles between oral contraceptive users and non-users (see Figure 4A). The mean plasma composition of the oral contraceptive population in controls was as follows: oral contraceptive users: fII 106%, fV 112%, fVII 118%, fVIII 124%, fIX 115%, fX 118%, AT 95%, TFPI 68%; non-users: fII 102%, fV 123%, fVII 104%, fVIII 117%, fIX 92%, fX 96%, AT 100%, TFPI 86%. As reported previously [15], when the factor levels are compared between the groups differences are seen in fIX, fX, AT and TFPI. When we evaluated the influence of the ensemble of plasma composition on the fXa generation output, we determined that that the increased fXa generation in women on oral contraceptives was primarily driven by differences in the levels of fIX and TFPI (Figure 6A). When the plasma composition accounting for the thrombin generation profiles of oral contraceptive users was similarly evaluated, the collective alteration of four factors (fIX, TFPI, AT and II) to the non-users values was required to fully normalize the thrombin generation output to that of the non-users (Figure 6B). Thus the minimal set of factors required to normalize fXa generation between the two populations is not equivalent to the minimal set required to normalize thrombin generation.

## Discussion

In this study we showed that there is a large variation in predicted fXa generation in an apparently healthy population and in individuals with a known DVT. The maximum rates and levels of fXa generation occur over a 10- to 12- fold range, respectively in both populations. This variation is larger than that observed with thrombin (3–6 fold) [21] and demonstrates that fXa generation is potentially a phenotypic characteristic and suggests that it may be useful as an individual marker for predicting thrombotic risk. The ORs for fXa generation are comparable to those calculated from the computational analyses of thrombin generation.

Factor Xa generation discriminated well between defined clinical risk subgroups. Most notably, healthy women on oral contraceptives showed >60% increase in some fXa parameters compared to women not on oral contraceptives. In addition, individuals with similar thrombin generation profiles displayed differing fXa generation profiles, and there appeared to be a linkage between the extent of the differences in the fXa generation profiles and the timing of thrombin generation in these pairs of individuals. Analysis of simulated fXa generation appears to be a more sensitive discriminator than simulated thrombin generation among individuals. In addition, these studies were performed with a Tf-initiator concentration of 5 pM to reflect empirically validated data [13]. Additional discrimination may be possible when outputs are evaluated at varying Tf concentrations; further studies are warranted to elucidate this effect.

Thrombin generation and fXa generation appear to have a similar ability to identify individuals at risk for DVT, and can tell us about the most thrombotic segment of the population. However, it is not a tool for predicting which individuals in the less thrombotic group (90% of the population) will transition to a thrombotic state. A measure of procoagulant capacity that shows a wider range of variation (fXa vs. thrombin generation) across an entire population, in principle, may have a stronger potential to prospectively identify individuals who will transition to a procoagulant state. At the peak of prothrombinase activity, the concentration of active fXa participating in prothrombinase represents less than 0.004% of the zymogen fX [8].

Utilizing empirically validated mathematical modeling, investigations and comparisons into protein interactions, intermediates or complex formations can be accomplished to develop a broader view of the dynamics associated with the coagulant response. In this study, these simulations account for all the plasma pro- and anti-coagulant proteins of the Tf-pathway that are both empirically validated and show correlation to clinical events and other clinical assays. Currently, these simulations do not include the contribution of the anticoagulant protein C pathway, the contribution of platelets, the contact pathway or the vasculature. In general, our theoretical approach allows us to evaluate the impact of each individual factor to the overall fXa or thrombin generation profile which may not be obvious using standard multivariate methods. We have previously shown that it is the integration of an individual's blood composition data at any one time into an assessment of that individual's thrombin generation potential that can be used to discriminate between various hemostatic states [15], [21], [23] and that changes in any single input value alone might not have any significant influence on the overall dynamics [16], [22], [24], [25]. Processing the input concentrations for each subject and performing simulations gives predictive capability beyond the original input concentrations.

Since thrombin is the key enzyme in Tf-initiated coagulation, but requires fXa formation and function for its generation, targeting fXa generation for analysis should increase our knowledge regarding the results that are seen in thrombin generation profiles. This is the first study that we are aware of that compares fXa generation to thrombin generation in the same population. To date, available diagnostic methods for fXa generation include chromogenic, immunological and clot based assays. Recently, a large focus has been on developing better methods for detecting fXa generation with increased sensitivity [26]–[28]. This focus has mainly been due to the development of anticoagulants targeting fXa generation and the need to monitor the efficacy. There is a greater potential for creating new targets for evaluation of fXa dynamics through simulations than what can be determined by empirical experiments, such as tracking fXa formed through either the intrinsic or extrinsic complex over time as a discriminator between subjects with high sensitivity.

The most dramatic separation of fXa generation profiles in was between users and non-users of oral contraceptives in control premenopausal women. The increased rate and ultimate levels of fXa generated suggests an extremely primed procoagulant response. The OR was similar to thrombin generation in this group [15], [21], being undefined for MaxR at a 90^th^ percentile and was 15.7 for MaxL. These data are consistent with the established link between oral contraceptive use and thrombotic risk [29], [30]. Many efforts are currently under way to decrease the risk of thrombosis seen in women on oral contraceptives [31], [32]. Previously, we have shown that women on high dose hormones, undergoing *in vitro* fertilization, had increased thrombin generation profiles [16] and that this effect on thrombin generation was in part due to alterations in plasma factors fVIII, TFPI and AT. In this study, we determined that the increases in fXa generation seen in oral contraceptive users were due to elevated levels of fIX and decreased levels of TFPI.

There is considerable interest in developing methods of analysis that relate an individual's thrombin generation profile derived directly from Tf-initiation of their plasma sample to their current or future risk of thrombosis [33]. Thrombin generation in apparently healthy individuals has been shown to be an individual phenotypic characteristic using either empirical approaches [34] or plasma composition based computational modeling [15]. In examining thrombin generation in healthy populations, there are clearly outliers, individuals whose thrombin profiles look like individuals with defined defects (*e.g.* factor deficiencies or factor over expression). These outliers appear to be obvious candidates in efforts to identify at risk individuals in the healthy population. However, most healthy individuals have relatively similar thrombin generation profiles, whether thrombin formation is evaluated by empirical or computational methods. The significance of small differences in these thrombin profiles is unclear, in part because an operational definition establishing what magnitude of difference is required to signal a consequence to an individual's hemostatic balance, and thereby his/her immediate or long term health, is lacking. An alternative approach for discriminating among these “thrombin similar” individuals may be to use additional analyte(s). This study potentially identifies the utility of fXa generation as a segregating tool for investigating groups of such individuals.

One limitation of the computational approach is the expense of factor analysis. We have previously reported that differences in thrombin generation between some populations may depend only on a subset of factors, all varying within the normal range [16], [22]. This suggests the possibility that specific hemostatic states may have a unique factor dependence thus reducing the expense in the context of a routine screening procedure. Data from this study shows an instance where effectively capturing differences between populations using evaluation of simulated fXa generation depends on a smaller subset of factor level measurements than when simulated thrombin profiles are used as the discriminator to evaluate the same populations. To the extent that further studies confirm the utility of fXa generation in stratifying individuals by clinical characteristics relevant to cardiovascular health, the reduced requirement for factor analyses improves the feasibility of the general approach. In addition, in this case-control study, blood was drawn after the event. We cannot be certain that our findings represent the situation just before the patients first deep vein thrombosis. The case population may have inflammatory reactions, which could affect coagulation assays. A previous publication on CRP and fVIII do not suggest this is the case in this group [35].

Utilizing plasma composition based modeling we can begin to develop individual blood response profiles that can be used to evaluate how an individual responds to varying triggers (*e.g.* Tf-stimulus, antithrombotics, oral contraceptives). These approaches can ultimately aid in the development of individualized therapies. These studies illustrate the importance of fXa specifically and prothrombinase as a therapeutic target.
